# Mental health disorders in child and adolescent survivors of post-war landmine explosions

**DOI:** 10.1186/s40779-015-0052-3

**Published:** 2015-11-13

**Authors:** Mohammad Ali Hemmati, Hamid Shokoohi, Mehdi Masoumi, Shahriar Khateri, Mohammadreza Soroush, Ehsan Modirian, Mahtab Poor Zamany Nejat Kermany, Maryam Hosseini, Batool Mousavi

**Affiliations:** Janbazan Medical and Engineering Research Center (JMERC), Tehran, Iran; Department of Emergency Medicine, George Washington University, Washington, DC USA; Emergency Department, Medical Faculty, Qazvin University of Medical Sciences, Qazvin, Iran; Anesthesiology and critical care Labbafinejad Hospital, Medical University of Shahid Beheshti, Tehran, Iran; Community and Preventive Medicine, Janbazan Medical and Engineering Research Center (JMERC), Tehran, Iran; NO.25, Farrokh St., Moghaddas Ardebily Ave., Chamran Highway, Tehran, Iran

**Keywords:** Landmine, Child survivors, Depression, Anxiety, Iran

## Abstract

**Background:**

To describe the mental health status of 78 child and adolescent survivors of post-war landmine explosions.

**Methods:**

Child and adolescent survivors of landmine explosions who were younger than 18 years old at the time of the study were identified and enrolled in this study. The mental health status of the participants was assessed by general health assessment and psychiatric examinations. Psychiatric assessment and diagnosis were undertaken using the Diagnostic and Statistical Manual for mental disorders (DSM-IV) criteria. A psychiatrist visited and interviewed each survivor and identified psychiatric disorders.

**Results:**

Seventy-eight child and adolescent survivors with a mean age of 16.11 ± 2 years old were identified and agreed to participate in the study. The mean age of the victims at the time of injury was 8.2 ± 3.12 years old (range 2–15). Thirty-seven (47.4 %) of the adolescent survivors suffered from at least one psychiatric disorder. Twenty-nine survivors (37.1 %) were newly diagnosed and needed to start medication and psychiatric treatment. The most common findings were anxiety disorders (34.6 %), including posttraumatic stress disorder (PTSD) in 20 (25.6 %), and generalized anxiety disorder (GAD) in 7 (9 %) subjects. Mild-Moderate depression was found in 5 (6.4 %) subjects. No personality disorders were observed, and two patients suffered from mental retardation. The study results revealed a significant association between age of casualty, duration of injury and limb amputation, and types of psychological disorders.

**Conclusion:**

Child and adolescent survivors of landmine explosions had a high prevalence of psychiatric disorders.

## Background

Landmines and unexploded ordnance (UXO) are common threats to civilians’ safety in the years following wars in mine-affected countries. According to the International Campaign to Ban Landmines, an estimated 15,000 to 20,000 people are killed or injured by landmines annually, with children accounting for one in every five victims [1, 2]. In a recent report by Landmine Monitor in 2012, it was estimated that, on average, more than one third of the accidents involving civilian landmines and explosive remnants of war occur with children [3].

Children are often attracted by the intriguing and colorful appearance of landmines and explosive remnants of war, and they might pick them up believing that they are toys. Despite all efforts of Child Friendly Spaces (CFSs) to train children to recognize and avoid unsafe areas and to undertake protective measures when they find war remnants, a large number of children are the victims of landmine injuries [4]. Current studies are focused on war veterans and combatant victims, despite the majority of modern warfare hazards threatening civilians more than soldiers. An association between war experiences and increased levels of mental disorders several years later has been previously confirmed [5].

In the aftermath of the Iraq-Iran War (1980–88), many civilians and children were affected and injured by landmines and UXO explosions along the Western and Southwestern borders of Iran. According to 2014 registry data, it was reported that 8,243 casualties (2,519 killed; 5,723 injured; one unknown) due to landmine explosions were identified in Iran between 1988 and 2013. Additionally, according to a report from the United Nation, it is estimated that approximately 10,000 casualties as the results of landmine and UXO explosions have occurred in the five Western and Southwestern border provinces of Iran after Iraq-Iran War [5].

The psychosocial impacts of injuries in this group of victims can range from mild stress reactions to problems such as anxiety, depression, substance abuse and posttraumatic stress disorder (PTSD), especially in child and adolescent survivors [6]. The rate of mental disorders among civilians in many war-affected countries in recent years has been reported at more than 30 % [5]. Studies of mental disorders in survivors of landmines have been rare, and there have been no studies assessing psychological disorders in children and adolescents who were landmine victims. Difficulty in relationships and daily functioning, social stigmatization, rejection and unemployment are common among adolescent survivors [8]. Data from landmine victims with traumatic amputation in six affected countries indicated that psychological recovery was greatly influenced by the following factors: the individual’s resilience characteristics, social support, medical care, the economic situation and societal attitudes toward people with disabilities [9].

In this study, we report the prevalence and types of psychiatric disorders among children and adolescents affected by landmines and UXO explosions following the Iraq-Iran war (1980–88).

## Methods

### Survey design and participants

Our study was a cross-sectional study focused on Iranian children and adolescents with landmine injuries. These victims were exclusively from the five Western and Southwestern provinces of Iran: Western Azerbaijan, Kurdistan, Kermanshah, Ilam, and Khuzestan. The subjects consisted of landmine victims who were younger than eighteen years old at the time of study. All of the victims were receiving the support of the Veterans and Martyr Affair Foundation (VMAF) of Iran. Written informed consent was obtained from all parents. A psychiatrist interviewed and evaluated each participant through a 10–20 min interview. This research protocol has been submitted for consideration, comment, guidance and approval to the research ethics committee of VMAF and accepted before the study begins. 

### Instrument

The demographic checklist included current age, age at time of injury, sex, education, location, duration of injury, type of injury and insurance coverage. In the second part, mental status examinations were performed based on psychiatrist observations, including appearance, temperament, affect, language, perception, illusion, thinking, orientation, long-term memory, short-term memory, judgment, insight and reliability. The presence of psychiatric disorders was determined by means of items corresponding to the multi-axial system of the DSM-IV (Diagnostic and Statistical Manual for mental disorders) criteria [10]: Axis I relates to a principal disorder that requires immediate attention, Axis-II consists of any personality disorder that might be shaping the current response to the Axis I problem, Axis III indicates any medical or neurological problems that might be relevant to the individual’s psychiatric problems, Axis-IV consists of the major psychosocial stressors that the individual has faced recently, and Axis-V includes the “level of function” that the individual has attained at the time of assessment. Axis III was not necessarily evaluated during the psychiatric interview but all of the participants were positive on this axis and were evaluated by other medical providers. Additionally, Axis IV was not examined due to the young age group.

### Data analyses

The Statistical Package for the Social Sciences, (SPSS 22.0, IBM SPSS Incorporated; Chicago, Illinois USA), was used for the statistical analysis. The data are presented as the means ± standard deviations. Pearson’s correlation coefficient and the chi-square test were used to examine the relationships between different types of psychological disorders and socio-demographic characteristics in this study. Means are depicted inside 95 % confidence intervals, and the alpha value was set at 0.05.

## Results and discussion

### Results

There were 78 children and adolescents, including 67 (85.9 %) male and 11 (14.1 %) female subjects, who participated in this study. The age of the study group ranged between 9 and 18 years old, with a mean of 16.1 ± 1.9 years old. At the time of injury, most of the participants were between 2 and 15 years old (89.5 %), and the mean age at the time of injury was 8.27 ± 3.12 years old. Sixty-three percent of the victims continued their educations after injury and 37 % quit their school training immediately or over the years after the injury. The socio-demographic characteristics are presented in Table 1. The mean interval between the injuries and the interviews was 7.86 ± 3.14 years.

**Table 1:** Socio-demographic characteristics of child and adolescent survivors of landmine injuries (*n* = 78)

Variables	Classification	Frequencies (%)
Gender		
	Male	67 (85.9)
	Female	11 (14.1)
Province		
	Kurdistan	29 (37.2)
	Kermanshah	25 (32.1)
	West Azerbaijan	13 (16.7)
	Ilam	7 (9.0)
	Khuzestan	4 (5.1)
Age of casualties *(At the Time of Injury)*		
	<5 years old	6 (7.7)
	5–9 years old	45 (57.7)
	10–14 years old	25 (32.1)
	>14 years old	2 (2.6)
Education after casualty		
	Yes	49 (63)
	No	29 (37)
Location		
	Urban area	31 (39.7)
	Rural area	47 (60.3)

Classification of the adolescents according to the type of injury showed that most of injuries led to amputation (52.6 %), and 25 (32.0 %) victims suffered from multiple physical injuries (Table 2) [11].

**Table 2:** Frequency distribution and types of injuries among child and adolescent survivors of landmine injuries

Type of injury	Frequencies (%)
Amputation	21 (26.9)
Spinal cord injury	3 (3.8)
Eye injury	7 (9.0)
Ear problem	4 (5.1)
Two injuries	18 (23.1)
Three injuries	5 (6.6)
Four injuries	2 (2.6)
Other problems	18 (23.1)
Total	78 (100)

**Fig. 1:**
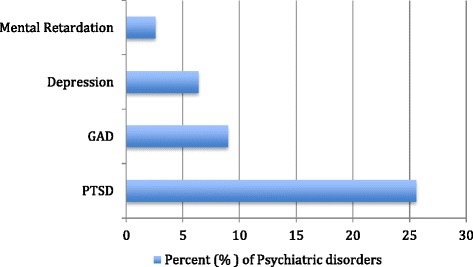
Frequency distribution of child and adolescent survivors with psychiatric disorders (*n* = 78). *PTSD = post-traumatic stress disorder, GAD= generalized anxiety disorder*

Nine (12 %) victims previously visited hospitals due to psychiatric disorders. Psychiatrists assessed thirteen mental health elements, as shown in Figure. 1. While more than half of the participants were abnormal in temperament, all were completely normal in five aspects: perception, illusion, thinking, insight and reliability. The abnormal characters were as follows: temperament in 42 (54.6 %), appearance in 6 (7.8 %), short-term memory in 5 (6.5 %), long-term memory in 3 (3.9 %), affection in 2 (2.6 %) and 1 (1.3 %) in language, orientation and judgment each.

The study showed that 37 (47.4 %) victims were affected by psychological disorders: anxiety disorders in 27 (34.6 %), mood disorders, including mild to moderate depression, in 5 (6.4 %), cognitive disorders in 2 (2.6 %) and other disorders in 3 (3.8 %). The anxiety disorders included PTSD in 20 (25.6 %) and generalized anxiety disorder (GAD) in 7 (9.0 %) (Fig. 1).

Significant relationships were observed of psychiatric disorders on Axis I with age of casualty and duration of injuries (*χ*^2^_(0.95,169)_ = 246.53, *P* = 0.03 and *χ*^2^_(0.95,52)_ = 69.83, *P* = 0.05, respectively). Landmine casualties at the age of 5 to 9 years old caused approximately one third of the mental disorders (40.9 %, *n* = 18). However, psychiatric disorders due to landmine incidents at younger than 5 years old (66.7 %, *n* = 4) and older than 9 years old (60.0 %, *n* = 15) were also quite common. In addition, the prevalence of mental disorders was inversely related to the duration of injury. Participants with anxiety or depression diagnoses totaled 8 (25.0 %) girls and 24 (75.0 %) boys, with a significant difference between genders (*χ*^2^_(0.95,4)_ = 13.031, *P* = 0.01). A more detailed analysis showed that two-thirds (63.4 %, *n* = 7) of girls suffered from anxiety disorders, while fewer than one third (29.8 %, *n* = 20) of boys were affected by this complication. Similar proportions were observed for depression in boys (5.0 %, *n* = 4) and girls (9.1 %, *n* = 1). Additionally, no significant relationships were found of age, age at the time of injury, education and disability rate with PTSD. The same results were observed in terms of other psychiatric disorders (*P* > 0.05). The chi-square test showed a significant relationship between amputation and type of psychological disorder (*χ*^2^_(0.95,12)_ = 28.85, *P* = 0.004), with more than one third (*n* = 14, 37.8 %) of amputees diagnosed as anxious.

**Fig. 2:**
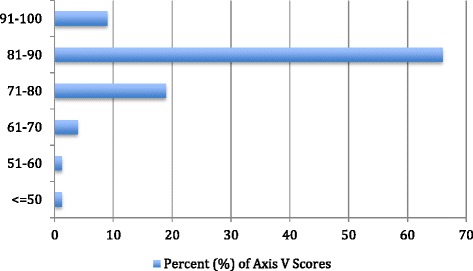
Frequency distribution of the axis V scores among child and adolescent survivors (*n* = 78)

Regarding Axis II, two patients (2.6 %) with mental retardation were detected. Axis III was positive for the entire group because all of the subjects had one or more physical injuries that were caused by landmine explosions. Regarding Axis V, only seven patients (9 %, *n* = 7) were normal and received the maximum score (91–100) (Fig. 2). More than two-thirds had scores of 80–90, indicating few or no symptoms, good functioning in several areas and no more than everyday problems or concerns. One of the new mental patients, with a score of 41-50, showed serious problems in school functioning and required educational support and counseling. The only person who had a history of psychiatric hospitalization had a score of 51–60 on Axis V. He expressed an anger disorder and limited affect, with impaired long- and short-term memory, orientation and judgment.

Thirty-eight (48.7 %) participants were mentally healthy, and 29 (37 %) required psychiatric treatment. Based on the analysis of DSM-IV diagnoses by psychiatrists, the following treatments were advised. Among the previously diagnosed participants, 3 (3.8 %) were advised to continue the previous therapy, 5 (6.4 %) needed to change their medications and psychotherapy, and 1 (1.3 %) was recommended to start psychotherapy and medications; among the victims never treated before, 5 (6.4 %) required pharmacotherapy, 12 (15.4 %) psychotherapy, 1 (1.3 %) cognitive behavioral therapy, 3 (3.8 %) psychological counseling, and 8 (10.2 %) were advised to start medications and psychiatric consultations.

## Discussion

For the first time, we estimated the number and type of mental disorders in adolescents affected by landmine and explosive remnants. More than one third of the survivors were new cases who had never been treated before and who needed to begin the treatment process. According to the previous literature, landmines have been the most common cause of war-related amputation in the past five decades [12]. Our findings supported the previous results and, in addition, showed that the type of injury caused by mine explosions could affect the type of mental disorder. Therefore, amputation was generally accompanied by mental disorders. Since the end of the Iran-Iraq War, landmines have caused devastating injuries in civilians that have often been fatal [2]. Despite all of the medical care, trauma-related issues and mental health problems among the survivors are quite typical, especially in children who suffer from permanent disability during their early years.

In this study, we described the mental health status of adolescents suffering from mine-explosion injuries. Almost half of the landmine survivors presented one or multiple psychiatric problems. The participants who previously had psychiatric outbreaks were found to require continued treatment due to insufficient therapy. In addition, more than one-third were patients who were never treated before and who were referred for psychotherapy, pharmacotherapy or both. Lack of adequate facilities and access to psychiatric department, as well as living in rural areas, could be the main cause of the detection of new mental patients. Anxiety and anger were the most common disorders among the subjects. The number of depressed adolescents was not considerable, which could have been due to the young age or due to depression in this age group requiring a different method of evaluation.

According to former studies, children are less likely to experience PTSD after trauma compared to adults, especially if they are younger than ten years of age [13]. However, in contrast with previous studies, the results of this study showed that adolescents with physical injuries developed a higher frequency of PTSD, which is more common in amputees following combat or accidental injury, whereas general rates of PTSD are 20 % to 22 % in cohorts without amputations [14]. In the current study, the higher rate of PTSD indicated that amputation and disability in daily routine tasks cause a variety of mental disorders, especially PTSD. Our findings reflected those reported of PTSD among landmine victims in other countries [15].

South Asian mine survivors, one quarter of whom are children, showed anxiety disorders, depression and PTSD at a prevalence of 80 %, 73 % and 71 %, respectively [16]. It was found that amputation severely changes the social life and free time activities of persons who were young at the time of amputation [17]. The prevalence of mental disorder estimates based on the DSM-IV in Iranian veterans with bilateral upper limb amputations was reported at 50.9 %, with the most frequent being temperament (18.4 %) and anxiety (14.6 %) [18]. However, the present study exclusively examined adolescents younger than 18 years old, and the results came entirely from this age group. Nevertheless, there was a similarity between the current results and those of previous studies. In contrast, the quality of life of Iranian adolescents (14–19 years old) who were survivors of landmines was evaluated at lower than normal levels, which confirmed the results obtained in this study [19]. Given that there have been no studies of coping mechanisms in affected children and teens in Iran, further research in this area seems essential. Our findings confirmed that PTSD was the most common complication encountered after mine blasts in adolescents, and the symptoms increased over time. Additionally, we found gender (female) to be a risk factor for developing more psychiatric disorders. Unfortunately, landmines and unexploded ordnances remain in the poor and rural areas, and because they live in these areas, blast victims are a considerable distance from health and psychiatric care centers. Obtaining higher education, better employment and opportunities for social interaction can be a great challenge for young injured survivors. To provide mental health services, psychiatric care after traumatic events and basic mental health information are essential. Counseling could help young landmine victims to learn new ways of thinking, to practice positive behaviors and to undertake active steps to move beyond their symptoms.

## Conclusion

This study was the first to assess the mental health status of children and adolescents who have been the victims of landmine explosions. Based on our results, the frequencies of mental disorders in this study group are high, and psychiatric care is essential. Because most of the children in rural areas have to work on farms or in animal husbandry, the high frequency of mental disorders could suggest that the victims expressed hopelessness due to being unable to perform physical activities and help their families. Thus, periodic evaluation of the mental health of injured adolescents in underserved areas is necessary. Follow-up of the mental health of these patients, as well as similar studies and coping mechanism surveys, is proposed for future.

## Abbreviations

UXO: Unexploded ordnance
CFSs: Child Friendly Spaces
DSM-IV: Diagnostic and Statistical Manual for mental disorders
PTSD: Posttraumatic stress disorders
